# Understanding the mismatch between behaviour and development in a novel host-parasitoid association

**DOI:** 10.1038/s41598-018-33756-6

**Published:** 2018-10-24

**Authors:** Joanna K. Konopka, Danny Poinapen, Tara Gariepy, Jeremy N. McNeil

**Affiliations:** 1 Department of Biology, Western University, London, N6A 3K7 Ontario Canada; 2 London Research and Development Centre, Agriculture and Agri-Food Canada, London, N5V 4T3 Ontario Canada; 3 Preclinical Imaging Research Centre, Robarts Research Institute, Schulich School of Medicine and Dentistry, Western University, London, N6A 5B7 Ontario Canada

## Abstract

Foraging parasitoid females should preferentially oviposit on hosts most suitable for progeny development to maximize their fitness. However, the introduction of a new host species may disrupt the link between the reliability of the cues and the expected adaptive outcome of female choice, leading to an evolutionary trap. This mismatch between behavioural acceptance and lack of development exists for North American and European egg parasitoids (Scelionidae) that encounter invasive *Halyomorpha halys* in areas where this exotic host has recently established. To explain this mismatch, we utilized an L9 orthogonal array design to assess and rank the influence of several critical factors characterizing host resource (host species, egg age, egg status, and surface wash) on behaviour (acceptance, patch residence and patch exploitation) and development of North American native *Trissolcus euschisti* egg parasitoid. Our results indicate that the host egg age is most important for behaviour, but is least influential for development of the progeny. This study suggests that the maladaptive decision to oviposit in an unsuitable host is due to a mismatch between the cues that females use, and the subsequent expected outcome of this choice. Therefore, it is the relative importance of individual factors when assessed simultaneously that influences the decision-making of female parasitoids.

## Introduction

The preference-performance hypothesis postulates that female insects will oviposit in or on hosts most suitable for their offspring’s development^1^. This preference should be most evident when the progeny are physically constrained to a limited resource, such as a parasitoid female laying in/on a host that will serve as the sole food source for her progeny. The general behaviours of foraging egg parasitoid females culminating in oviposition have been well described^2–5^. The first steps, finding a suitable habitat and searching for a host, involve the chemical cues emitted by the hosts themselves and/or those associated with the host (e.g. host plants, infochemicals left by the ovipositing herbivore)^6–9^. Once a female locates a host egg mass, she assesses the suitability of this host by antennation and ovipositor probing^10–14^. If the host is found suitable, she then oviposits, also leaving an oviposition deterrent pheromone^2,15–17^.

At any step in this behavioural sequence, the female’s decision will be influenced by several factors. These factors include the female’s biological and physiological state (e.g. age, egg load, nutritional or mating status, previous oviposition experience), or the perceived quality of the egg mass or individual eggs within the mass (e.g. age, size, shape, chemical cues, parasitized state or presence of a competitor)^18–23^. Other factors such as genetic variability of the parasitoids (e.g. differences in receptor sensitivity or activity levels), status of the host eggs (i.e. if the host eggs are fertilized or viable), or environmental stochasticity under natural conditions, can also affect the female’s decision^24–26^.

The introduction of a new (exotic) host species into the system can add a further dimension to the complexity of host acceptance. An introduced new host may disrupt the link between reliability of cues and the subsequent adaptive outcome, possibly leading to an evolutionary trap, especially if previously reliable cues are no longer indicative of developmental success of the progeny^27–29^. Subsequently, the developmental outcome (performance) cannot be reliably predicted by the female’s choice of the host (preference).

In North America and Europe, native scelionid egg parasitoids (*Telenomus* and *Trissolcus*), are faced with this problem following the introduction of *Halyomorpha halys* (Stål) (Hemiptera: Pentatomidae; the brown marmorated stink bug) from Asia. These native parasitoids readily oviposit in *H*. *halys* egg masses, but their offspring rarely complete development^30,31^. So far, no clear explanation or mechanism has been proposed for this mismatch between behavioural acceptance by these females and lack of larval development, even though significant efforts are being made to characterize and understand the impact *H*. *halys* has on the native parasitoid communities following its introduction^32^.

The surface chemicals, age and physical state (fresh or frozen) of the egg, as well as the presence of intrinsic or extrinsic competitors, are known to influence the female acceptance behaviours when encountering *H*. *halys* egg masses^31,33–35^. Although such studies provide valuable information about this host-parasitoid system, they usually focus on evaluating one or two factors at a time. Yet, it is important to understand the relative importance and reliability of each cue at different steps leading up to oviposition and marking. An ideal approach would involve testing multiple factors simultaneously for better understanding of their relative importance in the different decision making steps taken by foraging females (i.e. what those decisions are being influenced by the most), and the subsequent development of their progeny. A full factorial design incorporating all factors that may influence parasitoid behaviour and development is tedious and time consuming, making meaningful interpretation of the results possibly difficult.

Therefore, a systematic and unbiased approach to screen and rank the most influential factors affecting measured outcomes (in our case parasitoid behaviour and development), while simultaneously reducing experimental variability is needed. An orthogonal array (OA) design^36,37^ is ideal to overcome many of the challenges posed by the host-parasitoid system involving *H*. *halys*. This method allows the determination of critical factors and their optimal combination that affect (maximize or minimize) measured response variables (e.g. behaviour and development), and the ranking of their relative importance, as successfully applied in molecular biology, agriculture, biotechnology, and more recently in insect behaviour^38–41^.

We use the OA method to assess and rank the influence of several factors characterizing the host resource (host species, age of the eggs, status of the eggs, and presence of egg surface chemicals). We regard these factors as critical to understand the discrepancy between the host acceptance behaviour of a common North American egg parasitoid, *Trissolcus euschisti* (Ashmead) (Hymenoptera: Scelionidae), exploiting stink bug host egg masses (including *H*. *halys*), and the ability of the parasitoids to complete development in those egg masses.

## Materials and Methods

### Insect colonies

Eggs of three stink bug species were used, obtained from colonies (26 °C, 70% RH, 16 L:8 D photoperiod), established using field collected adults from Hamilton and London (ON, Canada) in 2012, and restocked annually. Two native species, the polyphagous *Euschistus variolarius* (Palisot de Beauvois) and the predatory *Podisus maculiventris* (Say), and the exotic *H*. *halys* were selected. Adults of *H*. *halys* and *E*. *variolarius* were held in BugDorm mesh cages (45 × 45 × 45 cm; ~50 adults/cage) and fed an organic diet consisting of romaine lettuce, carrots, apples, dry peanuts and soybeans, supplemented with zucchini, celery, and green beans when available. Adults of *P*. *maculiventris* were kept in plastic buckets (height = 15 cm; diameter = 15 cm) and fed *Tenebrio molitor* L. larvae. Stink bug egg masses were collected daily from the cheesecloth provided as an oviposition substrate.

Colonies of *T*. *euschisti* and *Telenomus podisi* (Ashmead) were established from parasitoid adults emerging from sentinel egg masses exposed in London in 2016, and maintained at 24 °C, 50% RH, 16 L:8 D photoperiod. Colony parasitoids were kept in clear plastic cups (height = 4 cm; Ø = 9 cm), provided with a honey water solution and fresh *P*. *maculiventris* egg masses once a week for oviposition. Parasitized colony egg masses were kept separately until adult emergence and experimental females were selected upon emergence from those egg masses. Experimental female parasitoids were collected daily and held in the presence of males but no host egg masses, until being used in the experiments.

### Orthogonal array

We employed an L9 (3^4^) (four factors at three levels) orthogonal array (OA) design (Table 1) to determine the relative importance of different cues on oviposition decision and development of *T*. *euschisti* (Table 2), focusing on main effects only (i.e. no interactions). This method allows us to not only determine which factors have an effect on parasitoid behaviour and development, but also to rank the relative importance of those factors (i.e. their relative influence on the measured response variables). The added advantage of OA design compared to full factorial design is the reduced number of experiments to run (9 for an L9 OA design vs. 81 for a full factorial with the same number of tested factors). Since different interactions can exist between tested factors for each measured outcome variable, we decided to use OA design that puts less emphasis on those interactions. We focused on main effects only because our interest was in the overall effect of each factor on several different measured outcomes associated with female behaviour and progeny development assessed simultaneously, as opposed to in isolation.

**Table 1:** L9 standard orthogonal array indicating combination of factors (4 factors each with 3 levels) and experiments (9 independent ones) to be performed.

Experiment no.	Factor A	Factor B	Factor C	Factor D
1	1	1	1	1
2	1	2	2	2
3	1	3	3	3
4	2	1	2	3
5	2	2	3	1
6	2	3	1	2
7	3	1	3	2
8	3	2	1	3
9	3	3	2	1

**Table 2:** Factors (host species, age of eggs, status of eggs, and surface wash) and their respective levels for the no-choice tests of *T*. *euschisti* (Hymenoptera: Scelionidae) host acceptance and development on stink bug (Hemiptera: Pentatomidae) egg masses.

	FACTORS			
	Host Species	Age of eggs (days)	Status of eggs	Surface wash
LEVELS	*Podisus maculiventris*	3	parasitized	acetone
	*Halyomorpha halys*	2	frozen	water
	*Euschistus variolarius*	1	fresh	none

In our design (Tables 2 and 3), the factors chosen to investigate the effect of the host egg mass on the parasitoid choice included: host species (*P*. *maculiventris*, *E*. *variolarius*, and *H*. *halys*), age of the eggs (1, 2 and 3 d), status of the eggs (parasitized, fresh, and frozen), and surface solvent wash (70% acetone, dH_2_O, and no wash). Since all used parasitoids had been reared on *P*. *maculiventris* eggs, egg masses of this species (fresh, 1d old, unrinsed) served as control (Experiment 3, Table 3). Therefore, we expected this treatment to give the best performance both by foraging females and developing progeny.

**Table 3:** An L9 orthogonal design with factor combinations for the no-choice tests of *T*. *euschisti* (Hymenoptera: Scelionidae) host acceptance and development on stink bug (Hemiptera: Pentatomidae) egg masses.

Experiment	Host species	Age of eggs	Status of eggs	Surface wash
1	*Podisus maculiventris*	3	parasitized	acetone
2	*Podisus maculiventris*	2	frozen	water
3	*Podisus maculiventris*	1	fresh	none
4	*Halyomorpha halys*	3	frozen	none
5	*Halyomorpha halys*	2	fresh	acetone
6	*Halyomorpha halys*	1	parasitized	water
7	*Euschistus variolarius*	3	fresh	water
8	*Euschistus variolarius*	2	parasitized	none
9	*Euschistus variolarius*	1	frozen	acetone

The goal of the OA analysis is to identify factors that reduce variability in the outcome variables, by minimizing the effect of noise factors (factors which are not easily controlled) to subsequently identify optimal factor settings. This process was done by calculating a signal to noise ratio (S/N ratio) for each factor and level, to determine which of the four factors (host species, age of eggs, status of eggs, and surface wash) reduced the variability in the outcome variables (behaviour and development). The calculated difference between maximum and minimum S/N ratios for each response variable (Delta (Δ)) provides a quantification of the effect of each factor on each outcome variable, and is used to rank the influence of each factor on each response variable.

For egg mass acceptance, patch exploitation (number of eggs drilled, marked, and superparasitized), and development, calculation of the S/N ratios were done to maximize the response (larger the better) using equation 1 (higher values indicate higher host attractiveness and resource utilization). The response was minimized (smaller the better) for S/N of patch residence (time on egg mass, time drilling, time drilling/egg) using equation 2 (lower values indicate more efficient use of time on a resource),

1 $$S/{N}_{i}=-\,10\,log(\sum ({\bar{y}}^{2})/{N}_{i})\,$$

2 $$S/{N}_{i}=-\,10\,log(\sum (1/{\bar{y}}^{2})/{N}_{i})\,$$

where $\bar{y}\,=\frac{1}{{N}_{i}}{\sum }_{u=1}^{{N}_{i}}{y}_{i,u}\,$ is the mean; *i* = experiment number, *u* = trial number, and N_*i*_ = number of trials for experiment *i*.

### Experimental set up

Fresh stink bug egg masses were collected daily from cheesecloth (oviposition substrate), separated into smaller clusters (12 eggs/mass), attached with the substrate to square pieces of white cardboard (~1 × 1 cm) with clear non-toxic glue (Ross®, Canada), and held in individual small (5 × 1 cm) Petri dishes until needed. For each age category, egg masses were subjected to different treatments before being assayed using the OA design (Table 3):

(i) egg masses were either fresh, or frozen for 5 min at −80 °C approximately 30–60 min before testing.
(ii) to obtain the ‘parasitized’ egg state, individual fresh egg masses were exposed to *T*. *podisi* females (1 female/egg mass) from the colony 24 h before the assay. All exposures were carried out under a stereomicroscope to ensure all the eggs were parasitized.
(iii) egg masses were left unrinsed or were immersed three times (10 s each wash) in approximately 1.5 mL of either 70% acetone (removal of cues with kairomonal activity) or distilled water (partial removal of water soluble cues)^2,13,42^. The rinsed eggs were allowed to dry completely before use. Surface washes are meant to remove host-deposited chemical cues, and parasitoid marking chemicals (in the case of “parasitized” egg status).

Fifteen, 4 day old, mated, naïve *T*. *euschisti* were tested for each of the nine experiments (i.e. n = 15 females for each experiment) (Table 3), and each individual was used only once. All assays were performed in individual Petri dishes at 24 ± 1 °C, 50% RH during the first 8 h of the scotophase (active period of parasitoids). Each parasitoid female was observed under a stereomicroscope, and was removed once all eggs were parasitized, or when the female showed no further interest in the egg mass (i.e. no antennation or exhibition of egg mass guarding behaviour).

The following parameters were measured: (i) if the female accepted the egg mass (as indicated by arrestment behaviour and substrate drumming followed by oviposition), (ii) patch residence: time from the first contact until the female finished marking; (iii) total time spent drilling and time drilling per egg as measures of decision making once on a resource; (iv) number of eggs drilled (those into which a female inserted her ovipositor) and marked (those on which a female dragged her ovipositor following oviposition) reflecting host attractiveness and perceived suitability for development of the progeny; (v) the incidence of superparasitism, where a female oviposits and marks an egg twice, and (vi) host suitability: offspring survival to adulthood from the different egg masses.

### Statistical analysis

Patch residence data (time on mass, time drilling, and time/egg) were checked for normality using Shapiro-Wilks and Levene’s tests, and then analyzed by one-way ANOVA with Tukey’s post hoc test following a logarithmic transformation. All other data (proportion of egg masses accepted, proportion of eggs drilled, marked and superparasitized) were analysed by χ^2^ tests, with values for each cell calculated based on standardized residuals (crosstab analysis) and compared with Bonferroni-corrected p values. Statistical analyses of qualitative and quantitative data were carried out using SPSS (v. 24, IBM Corp. USA) statistical software. The impact factors (host species, egg age, egg status, and surface solvent wash) were ranked based on the L9 OA designed and analyzed in Minitab statistical software (v. 18 Minitab, Coventry, UK).

## Results

### Behaviour

**Figure 1:**
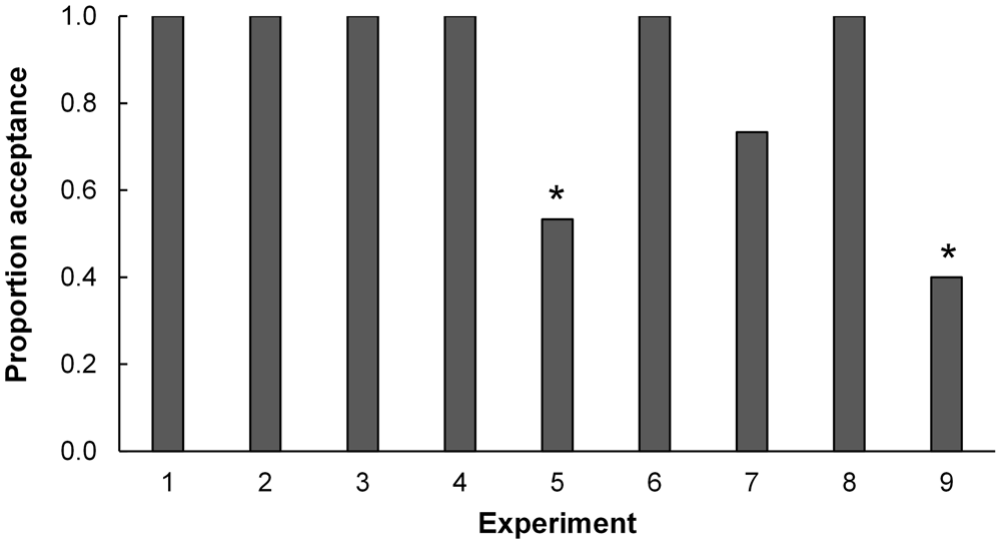
Acceptance of host egg masses by parasitoid females. Proportion of host egg masses accepted by *T*. *euschisti* females in nine experiments based on L9 (3^4^) orthogonal array (OA) design. Bars with asterisks (*) indicate experiments where the acceptance was significantly lower (p < 0.05) based on χ^2^ test.

*Trissolcus euschisti* females accepted *H*. *halys* and *E*. *variolarius* egg masses washed in acetone (Experiments 5 and 9 respectively), significantly less often as oviposition sites (53% and 40% respectively) (χ^2^_(8, N= 135)_ = 56.84, p < 0.001) than all other egg masses, including the control (Experiment 3; freshly laid non washed *P*. *maculiventris* eggs) (Fig. 1). Surface chemical cues and host species were the two most important factors affecting host acceptance. Surface chemicals were 2.5 times more important than egg age, which was ranked last (Table 4).

**Table 4:** Ranking of factors based on influence on *T*. *euschisti* egg parasitoid host acceptance, patch residence, patch exploitation, and progeny development from stink bug host egg masses.

	Parameter	Signal to noise ratio (Δ S/N)			
		Host species	Age of eggs	Status of eggs	Surface wash
Acceptance	Number of egg masses accepted	3.55 (2)	1.75 (4)	2.71 (3)	4.47 (1)
Patch residence	Total time on egg mass	0.60 (4)	1.99 (1)	1.59 (2)	1.19 (3)
	Total time drilling	1.23 (3)	2.25 (1)	0.75 (4)	1.30 (2)
	Time drilling/egg	1.02 (3)	1.85 (1)	0.66 (4)	1.79 (2)
Patch exploitation	Eggs drilled	0.10 (1)	0.09 (2)	0.04 (4)	0.07 (3)
	Eggs marked	1.48 (1)	1.21 (2)	0.56 (4)	0.95 (3)
	Eggs superparasitized	12.09 (3)	1.61 (4)	17.60 (1)	16.42 (2)
Progeny development	Proportion of *T*. *euschisti* emerged	10.00 (2)	6.05 (4)	20.52 (1)	7.02 (3)

Note: Delta (Δ) represents the difference between the maximum and minimum S/N for each factor at 3 levels (values represent the Δ, followed by a relative rank in brackets and ranks are assigned for each row individually). Factors with higher Δ have greater influence on a given measured variable (1 = most influence; 4 = least influence).

**Figure 2:**
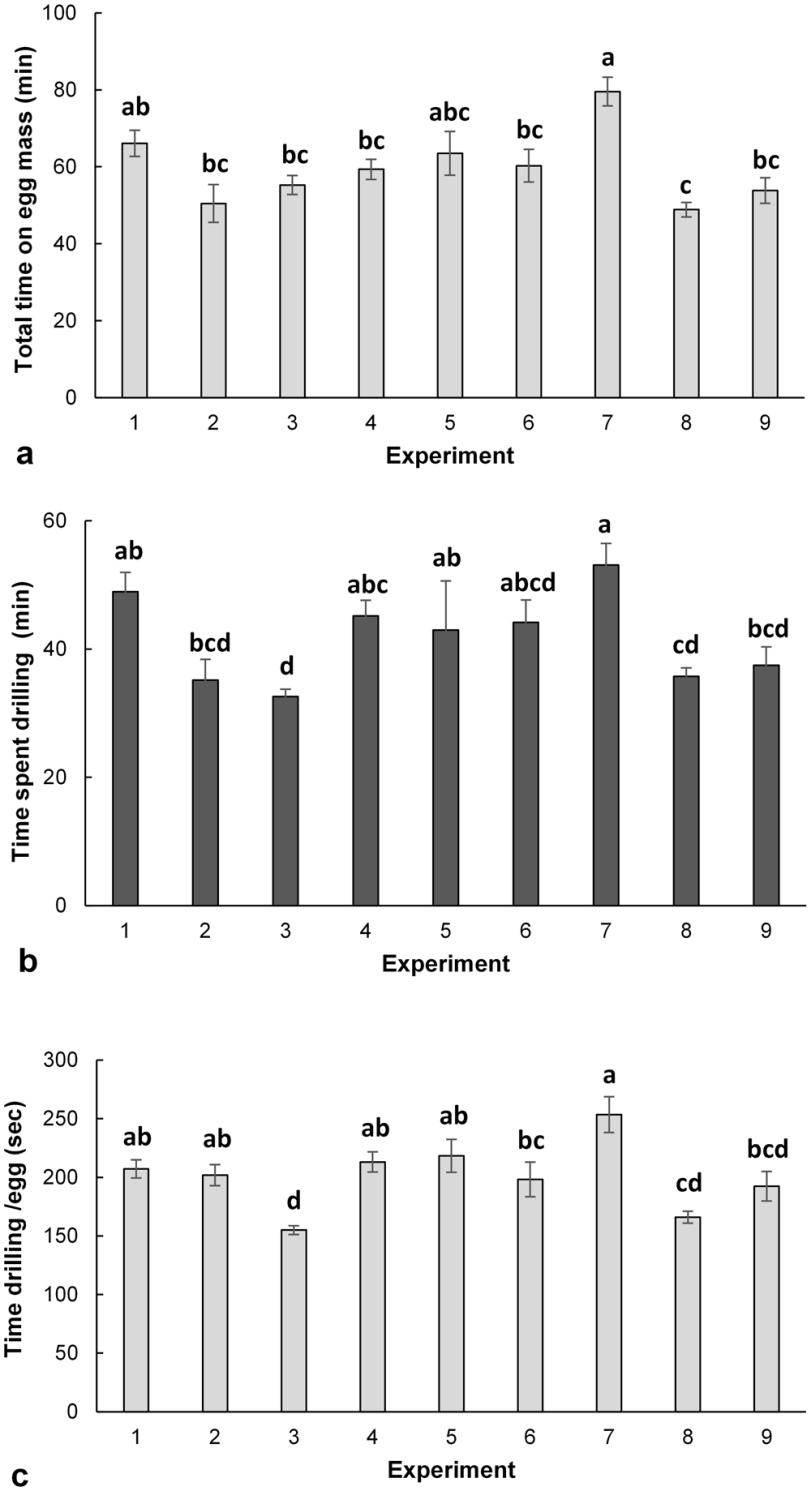
Behaviour (patch residence) of the parasitoids on host egg masses. Mean (±SE) (**a**) time spent on egg mass (**b**) total time spent drilling, and (**c**) time spent drilling per egg (for successful parasitization attempts) by *T*. *euschisti* females in nine experiments based on L9 (3^4^) orthogonal array (OA) design. Bars with the same lower case letter within each panel are not significantly different based on Tukey’s post hoc test (α = 0.05).

The estimates of patch residence, total time spent on an egg mass (F_(8, 105)_ = 6.16, p < 0.001; Fig., 2a), the total time spent drilling (F_(8, 104)_ = 7.22, p < 0.001; Fig. 2b), and the time to drill each egg (F_(8, 104)_ = 10.52, p < 0.001; Fig. 2c), followed similar patterns. The majority of time spent on an egg mass was allocated to oviposition (as indicated by drilling), and the remainder to unsuccessful parasitization attempts and surface antennation (those behaviours accounted for the time not spent drilling). Thus, it is not really surprising that the times spent on control egg masses (Experiment 3) were the shortest, while the longest times were observed on old, surface washed eggs of native stinkbug species. Age ranked as the most important factor for all three estimates of patch residence (i.e. total time, total time drilling, and total time drilling per egg), followed by surface chemistry (Table 4). Age was up to 3 times more influential than host species for total time on egg mass and egg status for time drilling.

**Figure 3:**
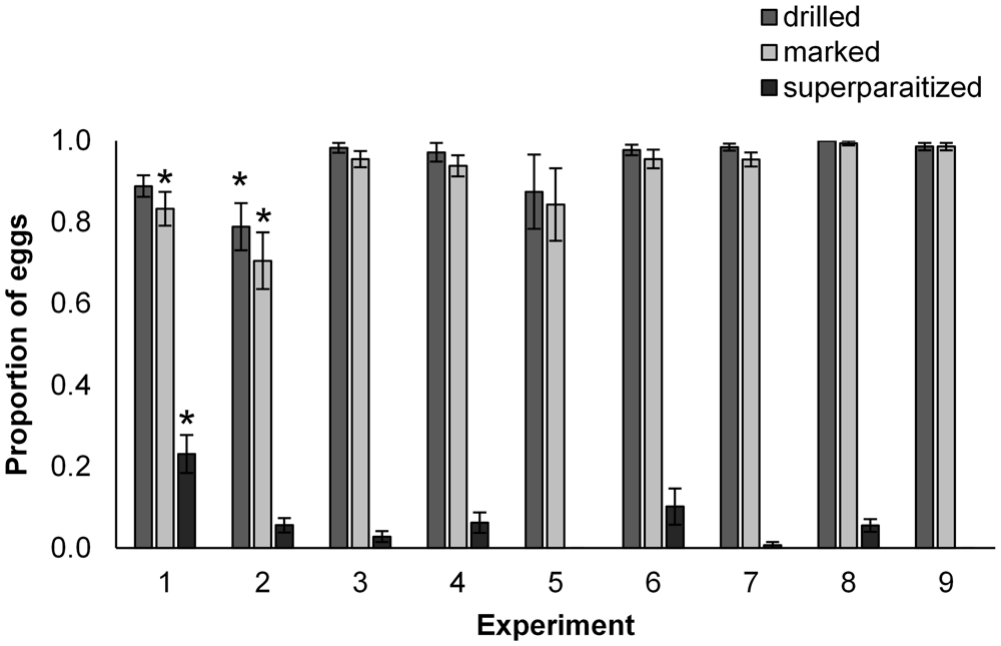
Behaviour (patch exploitation) of the parasitoids on host egg masses. Mean (±SE) proportion of eggs drilled, marked, and superparasitized by *T*. *euschisti* females in nine experiments based on L9 (3^4^) orthogonal array (OA) design, summarizing behaviour of the parasitoids on host egg masses. Bars with asterisks (*) indicate experiments where the means are significantly different (p < 0.05), among the different experiments based on χ^2^ tests for each measured outcome.

The proportion of eggs drilled and marked (on each prepared egg mass), and the incidence of superparasitism (a reflection of patch exploitation) did not vary among most treatments (Fig. 3). However, the response to parasitized and frozen, surface-washed, *P*. *maculiventris* egg masses (Experiments 1 and 2) differed significantly from all other treatments. The proportion of those eggs drilled was significantly lower in Experiment 2 (χ^2^_(8, N= 1380)_ = 118.18, p < 0.001), while the proportion marked was significantly lower in both Experiments 1 and 2 (χ^2^_(8, N= 1380)_ = 135.57, p < 0.001). Furthermore, the proportion of superparasitism observed was significantly higher in Experiment 1 compared with all other experiments (χ^2^_(8, N= 1295)_ = 92.61, p < 0.001). For both egg drilling and marking, host species and egg age were the two highest ranking factors (host species was 2.5 times more important than egg status), while egg status and surface cues were most important for superparasitism (egg status was 1.5 and 11 times more important than host species and egg age, respectively) (Table 4).

### Development

**Figure 4:**
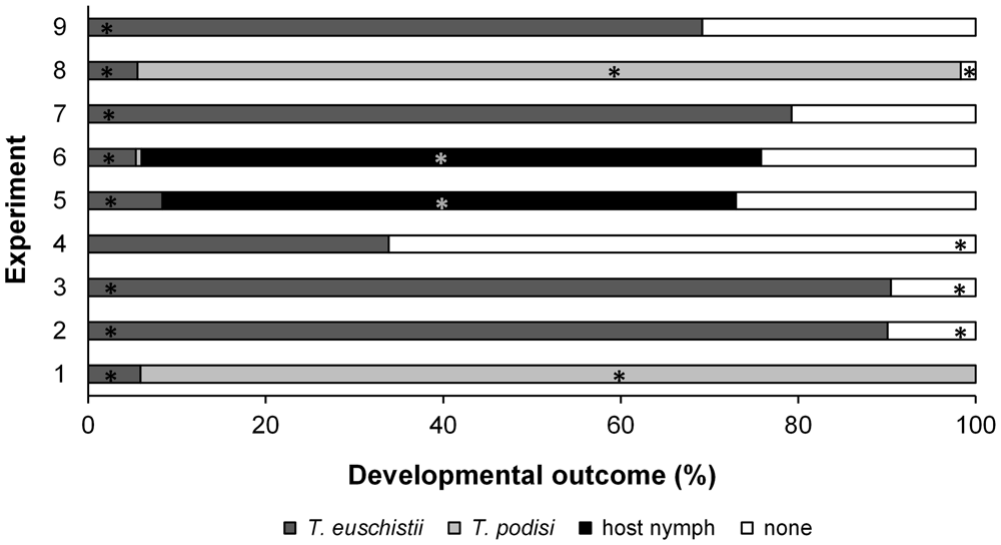
Development of the parasitoid on host egg masses. Mean proportion of *T*. *euschisti*, *T*. *podisi*, host nymphs (*P*. *maculiventris*, *H*. *halys*, or *E*. *variolarius*) or nothing emerging from egg masses parasitized by *T*. *euschisti* females in nine experiments based on L9 (3^4^) orthogonal array (OA) design. Asterisks (*) indicate proportions of each outcome that is significantly different from a mean proportion of that outcome across each of the nine experiments (based on χ^2^ tests with Bonferroni-corrections).

The proportion of each developmental outcome (i.e. *T*. *euschisti*, *T*. *podisi*, host nymphs, or no development) from parasitized egg masses differed among the treatments (χ^2^_(24, N= 1247)_ = 2230.7, p < 0.001). Stink bug nymphs only emerged from fresh and parasitized *H*. *halys* egg masses. In most experiments, there was a proportion of eggs that produced no parasitoids or hosts (Fig. 4). *Trissolcus euschisti* development depended on host species and status of the eggs (χ^2^_(8, N= 1248)_ = 722.30, p < 0.001), with 70–90% emerging from fresh (Experiments 3 and 7) or frozen (Experiments 2 and 9) native host eggs, and *P*. *maculiventris* being most suitable host for development (Fig. 4). However, *T*. *euschisti* completed development in <6% of native hosts previously parasitized by *T*. *podisi* (Experiments 1 and 8), with most multiparasitized egg masses producing *T*. *podisi*.

*Halyomorpha halys* eggs are clearly less suitable than native pentatomid species as <40% of frozen *H*. *halys* egg masses (Experiment 4) yielded *T*. *euschisti*, compared with 70–90% from frozen native host eggs (Experiments 2 and 9). Similarly, in both fresh *H*. *halys* eggs and those parasitized by *T*. *podisi* (Experiments 5 and 6), most eggs yielded stinkbug nymphs and <10% gave rise to parasitoids. Interestingly, only a small proportion of multiparasitized *H*. *halys* eggs resulted in parasitoid adults (mainly *T*. *euschisti*); in contrast multiparasitized eggs of native hosts (Experiments 1 and 8) yielded >95% parasitoid adults (mainly *T*. *podisi*). The most important factor for successful development of *T*. *euschisti* was egg status, followed by host species. Egg status was 2 times more influential than host species, and 3 times more important than both surface wash, and egg age (Table 4).

## Discussion

All cues that were investigated here provided foraging *T*. *euschisti* females with information leading up to oviposition, but their relative importance changed at different steps in the sequence of behaviours. The chemical cues from the egg surface and host species (undoubtedly inter-related) were the most important in the decision to approach and investigate a potential host. Without these cues (likely at least partially removed when the eggs were washed in acetone), a reduction in arrestment and substrate drumming was observed. The amount of time spent on the host egg mass and time spent drilling mainly depended on the age of the host eggs. Nevertheless, the actual decision to drill and mark the eggs following ovipositor probing was influenced by the host species identity. Following oviposition into the host, the likelihood of successful development of the parasitoid larvae was dependent on the egg status or viability of the host (fresh or frozen) and presence of competitors (parasitized egg state) (Table 5).

**Table 5:** Summary of decision making of foraging egg parasitoid (*T*. *euschisti*) on stink bug host egg masses based on the ranking of critical factors.

Behaviour/outcome	Mode of resource assessment	Decision to make	Decision most influenced by
Acceptance	Host searching	- Is this a potential host?	Surface chemical cues present
		- Should this resource be approached and investigated further?	
Patch residence	Antennation of the eggs	- How much time to spend on this patch?	Age of the host eggs
Patch exploitation	Ovipositor probing and oviposition	- Should more eggs be drilled?	Host species
		- Should eggs be marked?	
Progeny development	Post oviposition	- Can host defences be overcome?	Host viability and presence of competitors
		- Are there competitors present?	

Our study demonstrated that the behaviour and development of *T*. *euschisti* egg parasitoids are influenced by separate and distinct critical factors associated with the host egg resource. Although the predominant factor influencing parasitoid female behaviour once the female accepted the resource and started ovipositing was the age of host eggs, this factor was ranked as least important for the successful development of progeny. Similarly, the predominant factor influencing development of progeny (egg status) was ranked last among factors influencing behaviour. This observed reversed ranking of critical factors represents a mismatch between behaviour and development, and questions the reliability and relative importance of different cues for the foraging parasitoid females. The fact that parasitoid development, but not the behaviour of the foraging females, was mostly influenced by the presence of competitors and viability of the host (i.e. status of the egg), indicates that native egg parasitoids are making a maladaptive decision to oviposit in an unsuitable host. This decision is linked to a mismatch between the cues females are able to detect, and the subsequent expected adaptive outcome of their choice.

To maximize fitness gain, foraging parasitoid females can either increase the time in high quality patches (by spending more time assessing the suitability of a resource), or decrease the time spent per individual host within the patch once deemed acceptable^43^. This time-allocation was observed when *T*. *euschisti* was provided young, fresh, intact *P*. *maculiventris* eggs (see Figs 2 and 3, Experiment 3). Our results show that the age of the eggs is a very important cue for patch residence time (total time, time drilling) and patch exploitation (drilling and marking), indicating that information obtained once oviposition has been initiated affects the time spent on the egg mass. The finding that females spent the most time drilling the oldest eggs (as seen in Experiments 4, 5, and 7) of the native hosts with which they were familiar, is linked to the general decline in host quality with age, and relatively higher risk associated with parasitizing an older host (e.g. possible immune response of the developing host, difficulty in probing for small yolk volume, or not enough resources for completion of the development)^5,20,44^.

Our findings provide some explanation why eggs of exotic *H*. *halys* are accepted by native parasitoids as a host, even though they are unsuitable for progeny development. A reduction in host recognition and acceptance by *T*. *euschisti* was observed in acetone washed *H*. *halys* and *E*. *variolarius* egg masses, but not in those of *P*. *maculiventris*, suggesting that in the latter case some chemical cues were not removed by the solvent used. This finding indicates that there might be species-specific differences in volatiles mediating parasitization of pentatomid eggs^35^, and thus a chemical similarity with a successfully-exploited native species could explain why *H*. *halys* eggs are perceived by foraging native parasitoid females as suitable patches worth exploiting. Furthermore, once on the patch, the age of the eggs was almost as important as host species with respect to oviposition. Thus, there are sufficient cues to stimulate oviposition even though the host may be unsuitable for development of the progeny. Although Tognon *et al*.^45^ suggested surface chemicals from *H*. *halys* eggs inhibited parasitization by native North American egg parasitoids, our behavioural data do not support this idea, at least for *T*. *euschisti*. In fact, *T*. *euschisti* females are quite willing to lay eggs in viable *H*. *halys*, but are unable to complete development, just like their European counterparts^31,34^. Since we did not investigate or assess the actual chemical components from the host eggs, further research is needed to identify the infochemicals present and determine their relative importance.

Based on data from the current study and in the literature, *H*. *halys* is not a high quality host once an egg has been laid, with the viability of the host and presence of the competitors being the highest ranked factors for the successful development of the parasitoid. Freeze-killed eggs increase the developmental suitability for North American and European egg parasitoids on *H*. *halys*, compared to fresh eggs^31,34^. This improved developmental success does not occur in eggs of native species like *P*. *maculiventris*, where freezing provides no additional advantage to *T*. *euschisti*. Yet, the number of progeny produced from frozen *H*. *halys* eggs was still much lower, compared with frozen eggs of native hosts, suggesting that *H*. *halys* is inherently less suitable. Therefore, the presence of a developing host embryo, is not the only factor preventing parasitoid development in fresh *H*. *halys* eggs. The difference in the suitability of *H*. *halys* and native host eggs for parasitoid development could be related to factors such as the physiological environment and/or the quality and quantity of available resources. For example, the initial egg content composition, as well as the changes in nutritional quality might differ between *H*. *halys*^46^ and native hosts following freezing. Furthermore, in larger *H*. *halys* eggs, parasitoid larvae may be unable to consume the host tissue fast enough, such that the decomposition of frozen host tissues results in an unsuitable environment.

Although European *T*. *cultratus* is capable of producing some progeny from fresh *H*. *halys* egg masses, by acting as a facultative hyperparasitoid on *T*. *japonicus* when in interspecific competition^34^, this phenomenon was not observed when *T*. *euschisti* was offered stink bug eggs previously parasitized by *T*. *podisi*. In the case of *H*. *halys*, lack of facultative hyperparasitism might occur because *T*. *podisi* is unable to develop or kill the eggs of this exotic host, and future research should explore the potential of *T*. *euschisti* (or other native North American parasitoids) to develop as facultative hyperparasitoids on *H*. *halys* eggs following successful exploitation by *T*. *japonicus*. For native stink bugs (*P*. *maculiventris* and *E*. *variolarius*) (Experiment 1 and 8, Fig. 4), *T*. *podisi* was the dominant parasitoid reared from the egg masses, suggesting that *T*. *euschisti* do not act as a facultative hyperparasitoid on *T*. *podisi*. Since facultative hyperparasitism usually occurs during a very specific time window in the developmental process^47–50^, we did not observe it, given the short time interval between the first and second parasitizations in the current study.

The influence of host choice on parasitoid fitness is based on the premise that females make host selection using reliable cues that indicate past success, thus making their choice adaptive. From an ecological perspective, parasitoids are time- and egg-limited. The time and resources that parasitoids allocate to a resource patch will affect the number of progeny they are able to produce, and hence their fitness. The inability of the egg parasitoids to recognize hosts unsuitable for development of their progeny, whether this unsuitability is due to the host species (e.g. an exotic species, such as *H*. *halys*) or the egg status of a known host (e.g. parasitized *P*. *maculiventris*), results in wasted reproductive effort, both in terms of time (time sink) and resources (egg sink)^30,51,52^. A subsequent reduction in parasitoid populations could have both direct (e.g. increase in populations of native stink bugs) and indirect (e.g. increased apparent or direct competition) effects on trophic interactions between the stink bug and natural enemy communities^53,54^.

Therefore, from an evolutionary perspective, selective pressure to avoid unsuitable hosts^55^, or to overcome barriers to development in those hosts^56^ is likely high for parasitoids^30,32^. In the case of native parasitoids (including *T*. *euschisti*), there is considerable evidence that they do not avoid *H*. *halys* egg masses, in part because they provide cues similar to the native pentatomids with which they have co-evolved. The inability of parasitoids to discriminate between a suitable and unsuitable host resource would make *H*. *halys* an evolutionary trap^30^, unless the native parasitoids are able to overcome the barrier to successful development. While *H*. *halys* has only been present in North America since the 1990s^57^, three egg parasitoid genera (including *Telenomus*) have been recorded to have at least partial success on fresh *H*. *halys* egg under field conditions^58,59^. This partial success suggests that native parasitoids may have already started adapting to this novel *H*. *halys* host^32,60^. This progressive adaptation of North American parasitoids to *H*. *halys* could possibly be due to ecological and phylogenetic similarity of *H*. *halys* to the hosts that the parasitoids normally attack, thus shortening the time required for natural enemy recruitment^61^.

From a biological control perspective, native parasitoids may still provide limited control of *H*. *halys* via egg abortion^62^, as seen in the reduction in emergence following parasitization by *T*. *euschisti* (either alone or with *T*. *podisi*), even though the parasitoids were unable to complete development. However, in a short term, a classical biological control approach using Asian egg parasitoids (e.g. *Trissolcus japonicus*) that have co-evolved with *H*. *halys*^63^ or native eupelmid parasitoids (e.g. genus *Anastatus*) that are frequently reported from field collected egg masses^31,58^ would be more effective. In a long term, the interspecific interactions between introduced and resident parasitoids could change as native species adapt to the exotic host.

While the OA approach reduces the time and resources required to screen and determine relative importance (i.e. ranking) of critical factors, it has some limitations. First, the L9 design used here tests main effects only, and does not include possible interactions among factors. There is no doubt that interactions do exist^30,34^, so further experiments can be carried out using a full factorial design once the most important factors have been determined, as in the current experiments for each measured outcome (i.e. female behaviour and progeny development). Therefore, in our study, the OA serves as an initial screening method to minimize the amount of time and resources required to reach similar conclusion when using other methods^39^. Additionally, there are some constraints associated with specific levels of factors used in our study, which might affect the relative influence of those factors on the measured variables. For example, if wider range of host egg age was used, this factor might have stronger influence on female host acceptance.

Although this approach for determining the causes of the mismatch between behaviour and development in egg parasitoids is unconventional, our study enabled the ranking of certain critical factors. While individual factors may affect behaviour (acceptance, patch time allocation, host resource exploitation) and development of the parasitoids, it is the relative importance of individual factors when assessed simultaneously (as opposed to in isolation) that influences the decision making of the parasitoids. If the goal is to minimize host emergence and maximize natural enemy emergence (as would be necessary in a biological control program against *H*. *halys* using egg parasitoids), then utilizing the OA approach can significantly reduce the time and effort required to obtain critical information on the key factors affecting parasitoid behaviour and development. Consequently, this approach could provide a new way of interpreting host-parasitoid interactions (both from an ecological and evolutionary perspective), and enable more efficient screening of biological control agents, and their impacts on target and non-target organisms.

## Data Availability

The datasets generated and/or analysed during the current study are available from the corresponding author on reasonable request.
